# Harnessing the Immunomodulatory Properties of Bacterial Ghosts to Boost the Anti-mycobacterial Protective Immunity

**DOI:** 10.3389/fimmu.2019.02737

**Published:** 2019-11-22

**Authors:** Jieling Lim, Vanessa Hui Qi Koh, Sharol Su Lei Cho, Balamurugan Periaswamy, Dawn Poh Sum Choi, Maurizio Vacca, Paola Florez De Sessions, Pavol Kudela, Werner Lubitz, Giorgia Pastorin, Sylvie Alonso

**Affiliations:** ^1^Department of Microbiology and Immunology, Yong Loo Lin School of Medicine, National University of Singapore, Singapore, Singapore; ^2^Immunology Programme, Life Sciences Institute, National University of Singapore, Singapore, Singapore; ^3^Genome Institute of Singapore, Agency for Science, Technology and Research (A*STAR), Singapore, Singapore; ^4^Biotech Innovation Research Development & Consulting (BIRD-C), Vienna, Austria; ^5^Department of Pharmacy, Faculty of Science, National University of Singapore, Singapore, Singapore

**Keywords:** bacterial ghosts, tuberculosis, host-directed therapy, drug resistance, adjunct immune therapy

## Abstract

Tuberculosis (TB) pathogenesis is characterized by inadequate immune cell activation and delayed T cell response in the host. Recent immunotherapeutic efforts have been directed at stimulating innate immunity and enhancing interactions between antigen presenting cells and T cells subsets to improve the protective immunity against TB. In this study, we investigated the immunostimulatory properties of bacterial ghosts (BG) as a novel approach to potentiate the host immunity against mycobacterial infection. BG are intact cytoplasm-free *Escherichia coli* envelopes and have been developed as bacterial vaccines and adjuvant/delivery system in cancer immunotherapy. However, BG have yet to be exploited as immunopotentiators in the context of infectious diseases. Here, we showed that BG are potent inducers of dendritic cells (DC), which led to enhanced T cell proliferation and differentiation into effector cells. BG also induced macrophage activation, which was associated with enhanced nitric oxide production, a key anti-mycobacterial weapon. We further demonstrated that the immunostimulatory capability of BG far exceeds that of LPS and involves both TLR4-dependent and independent pathways. Consistently, BG treatment, but not LPS treatment, reduced the bacterial burden in infected mice, which correlated with increased influx of innate and adaptive effector immune cells and increased production of key cytokines in the lungs. Finally and importantly, enhanced bacilli killing was seen in mice co-administered with BG and second-line TB drugs bedaquiline and delamanid. Overall, this work paves the way for BG as potent immunostimulators that may be harnessed to improve mycobacteria killing at the site of infection.

## Introduction

Tuberculosis (TB) remains one of the most persistent diseases of the modern world, despite steady improvement in TB mortality and incidence rates in 2017 (1). Many factors contribute to the high burden of disease including poverty, HIV co-infection, limited vaccine efficacy and drug resistance (2, 3). As the development of vaccines with improved protective efficacy (4) and drugs with novel modes of action (5) has proven to be challenging and expensive, alternative solutions are urgently needed.

The main causative agent of TB, *Mycobacterium tuberculosis* (Mtb) is an intracellular pathogen that is capable of infecting a variety of cell types including epithelial, myeloid and lymphoid cell lineages. This pathogen has evolved numerous strategies to counteract, escape, subvert or delay the host protective immune responses. In innate immune cells, such as macrophages and dendritic cells (DC), Mtb hinders phago-lysosomal fusion (6), limits MHC antigen presentation (7), inhibits apoptosis (8), and dampens the migratory potential of DC (9). At the adaptive immunity level, Mtb-specific CD8 T cells were found to exhibit suppressed cytotoxic activity and proliferative ability due to impaired differentiation (10, 11). Importantly, Mtb also skews the protective Th1-mediated immunity toward Th2 responses by perturbing IFNγ signaling and inducing high IL-4 levels, which results in reduced iNOS activity, impaired apoptosis of infected cells, increased regulatory T cell numbers and greater iron availability to intracellular Mtb (12, 13).

Host-directed therapies (HDT) have been increasingly explored as alternative or adjunct TB treatment that focus on potentiating the host (immune) responses to improve mycobacterial killing (14, 15). Some notable examples include interferon (IFN) α or γ therapy (16–18), antibody-based therapy (19–21), metabolic pathways targeting approaches (22, 23) and therapeutic vaccination with non-pathogenic mycobacteria or Mtb fragments (24–26).

Here, we investigated the therapeutic potential of *Escherichia coli* bacterial ghosts (BG) against TB. BG are cytoplasm-free, intact bacterial cell envelopes that are obtained through the conditional expression of plasmid-encoded gene E from the bacteriophage ΦX174 (27). Integration of the 91 amino-acid polypeptide E in the bacterial envelope triggers a fusion process of the inner and outer membranes to form a transmembrane tunnel structure through which the cytoplasmic content is expelled driven by a proton-motive force (28, 29). To date, BG have been made from a variety of pathogens including *Escherichia coli* K12 (30), enterotoxigenic and enterohemaorrhagic *E. coli* (EHEC, ETEC) (31), *Helicobacter pylori* (32), *Salmonella typhimurium* (33), *S. enteritidis* (34), and *Vibrio cholerae* (35) for both veterinary and clinical vaccine purposes. BG have also been evaluated as drug delivery (36) and adjuvant (37) systems. Additionally, mucosal routes, including oral, aerosol and intranasal, have been deemed suitable for BG administration (38–41). The presence of various pathogen associated molecular patterns (PAMPs) in the cell wall of BG—lipopolysaccharide (LPS), peptidoglycan, glycolipids, flagellin, and lipoproteins—makes them potent activators of innate immune cells, which leads to the production of pro-inflammatory cytokines and bactericidal elements, such as reactive oxygen and nitrogen intermediates (ROIs and RNIs) (37, 42, 43). Furthermore, through their ability to activate DC, BG have also been shown to promote greater pathogen-specific antibody responses (40), increased T lymphocytes recruitment and proliferation with their associated cytokine production (39, 41, 44, 45).

In this study, the immunostimulatory properties of BG were assessed in the context of mycobacterial infection and our data demonstrate that BG can enhance mycobacterial killing and improve the efficacy of second-line TB drugs. With greater development to further boost the extent of killing, BG may represent a promising option for HDT.

## Methods

### Bacteria, BG, and Mammalian Cell Culture

Cultures of *M. bovis* BCG Pasteur strain (ATCC, 35734) were cultured in standing T25 flasks in Middlebrook 7H9 broth (Becton Dickinson Difco™, NJ, USA) at 37°C until OD_600nm_ 0.5–0.7 and then stored at −80°C in 7H9 and 25% glycerol (1st Base, Singapore). Mtb H37Rv were cultivated in 50 mL 7H9 rolling culture and processed as described above.

Bacterial ghosts (BG) prepared from *Escherichia coli* NM522, as previously described (30), were obtained from Biotech Innovation Research Development & Consulting (BIRD-C, Austria).

Bone marrow cells flushed out from femurs and tibia of 7–8 weeks-old female WT or TLR4 KO C57BL/6 mice were differentiated into macrophages (BMMO) over 6–8 days at 37°C, under 5% CO_2_ in complete differentiation medium containing DMEM (Gibco), 1X penicillin-streptomycin (Gibco), 1 mM sodium pyruvate (Gibco), 10% heat-inactivated fetal bovine serum (FBS) (Gibco), and 20 ng/mL murine M-CSF (R&D Systems) in HEPES (Gibco). Bone marrow derived dendritic cells (BMDC) were cultured in complete differentiation medium—RPMI (Gibco), 10% FBS, 0.05 mM β-mercaptoethanol (Gibco), 1 × glutamax (Gibco) with 20 ng/mL murine GM-CSF (Peprotech)—over 10–12 days. Media changes were conducted on 3rd, 6th, 8th, and 10th day of culture. BMMO and BMDC were maintained in antibiotic-free, complete medium containing 10 ng/mL murine M-CSF and 5 ng/mL murine GM-CSF, respectively after differentiation.

### *In vitro M. bovis* BCG Infection

BMMO and BMDC (5 × 10^4^ cells) were infected with *M. bovis* BCG at a multiplicity of infection (MOI) of 2 for 1 h at 37°C and 5% CO_2_. The cells were then washed once with 1 X PBS to remove extracellular bacteria. At indicated time points, cells were lysed by vigorous flushing with 0.1% Triton X-100 (USB Corporation). Cell lysates were serially diluted in Middlebrook 7H9 broth before plating onto 7H11 agar plates (Difco, BD) for colony forming unit (CFU) enumeration after 16–19 days incubation at 37°C and 5% CO_2_.

### LPS Extraction From BG and Quantification

BG (10^9^) lyophilized particles (17.8 mg) were resuspended in 2 mL of millicule water and pelleted by centrifugation. BG pellet was resuspended and lysed, before total LPS was extracted using the LPS Extraction Kit from iNtRON Biotechnology (South Korea), according to the manufacturer's instructions. Extracted LPS was then washed using 70% EtOH, before reconstitution in 10 mM Tris-HCl. The amount of LPS extracted from BG was quantified using Pierce™ Limulus Amebocyte Lysate Chromogenic Endotoxin Kit (Thermofisher Scientific, MA, USA). Results indicated a total LPS content of 7.5 × 10^−5^ EU/BG particle.

### BG, LPS, and IFNγ Treatments of Macrophages and DC

BMMO and BMDC were treated with BG at MOI 40 and 5, respectively or as indicated in the figure legends. Treatment with TLR4-specific lipopolysaccharide (LPS) from *E. coli* 0111:B4 strain (LPS-EB Ultrapure) (Invivogen; CA, USA), murine recombinant IFNγ (Gibco) were done at a concentration indicated in the figure legends.

### Mouse Experiments

Animal experiments were approved by the Institutional Animal Care and Use Committee of National University of Singapore (NUS) under protocol R15-1030 and were performed in the AALAAC-accredited animal facilities at NUS. 7–8 weeks old female Jackson C57BL/6 mice were purchased from InVivos (Singapore) and were intratracheally (IT) infected with ~10^6^ CFU of *M. bovis* BCG or 10^3^ CFU of Mtb H37Rv. Starting day 7 p.i. or as indicated in the figure legends, BG (10^6^ particles/mouse), normalized LPS treatment (75 EU/mouse corresponding to 75 ng) or vehicle (millicule water) were administered IT once weekly up to 4 consecutive weeks. Drug treatments started on day 7 p.i. INH was incorporated in drinking water of mice at 0.05 mg/mL for daily consumption. All other drugs were formulated in 20% D-α-tocopherol polyethylene glycol 1000 succinate (TPGS, Sigma-Aldrich) + 1% DMSO and were administered by oral gavage thrice weekly for 4 consecutive weeks at the following doses: Q203 (10 mg/kg), bedaquiline (BDQ; 15 mg/kg), delamanid (DLM; 15 mg/kg) and linezolid (LZD; 50 mg/kg). For combined therapy, mice were treated by oral gavage or IT on separate days to prevent excessive handling. Mice were monitored daily for weight loss. At specified time points, lungs from euthanized mice were harvested and homogenized on ice in PBS + 0.1% Triton X-100 with Halt protease inhibitor cocktail (Thermofisher Scientific). Appropriate dilutions of the lung homogenates were plated onto 7H11 agar (Difco, BD) for CFU determination after 16–19 days incubation at 37°C and 5% CO_2_. Alternatively, lung homogenates were spun down and the supernatants were collected for cytokine quantification. For lung histology, lungs from euthanized mice were inflated with 4% paraformaldehyde (PFA) and kept at 4°C overnight before H&E staining. For flow cytometry analyses, mice were euthanized at the indicated time points and harvested lungs were treated with collagenase D (Roche, Germany) for 1 h at 37°C and gently mashed through a cell strainer to obtain single cell suspensions before quenching with FACS buffer, containing 2% FBS, 2 mM EDTA in PBS.

### Flow Cytometry

Single cell suspensions from murine lungs were treated with red blood cell (RBC) lysis buffer and blocked with mouse Fc block (BD Pharmingen, Germany). Cells were stained for 30 min with Live/Dead eFluor780 Fixable Viability Dye (ThermoFisher Scientific) and the following panel of antibodies were used: (i) myeloid cells: CD45 BUV395 (BD), CD11b eFluor450 (Thermofisher Scientific), CD11c BV786 (BD), CD24 BUV496 (BD), Ly6C-Biotin and BV805-Streptavidin (BD), Ly6G PE-CF594 (BD), CD64 PE-Cy7 (Biolegend), Siglec F BV510 (BD), MHCII (IA/IE) PE (Biolegend, CA, USA), CD86 Alexa647 (Biolegend); (ii) pDC and T cell subsets: CD45 BUV395 (BD), CD11c APC (BD), Siglec H PE (ThermoFisher Scientific), Thy1.2 PB (Biolegend), CD4 BUV805 (BD), CD8a AlexaFluor488 (Biolegend), CD44 BUV737 (BD), CD62L BV605 (BD), CD45Ra BV786 (BD). Immune cell populations were defined according to the gating strategy shown in Figure S4.

For *in vitro* experiments, the antibodies used were as follows: (i) BMMO: CD11b eFluor450, F4/80 PerCP-Cy5.5, CD80 APC, CD86 PE, CD54 FITC, Live/Dead eFluor780 and (ii) BMDC: CD11c FITC, MHCII PB, CD86 PE, CD40 APC, CCR7 PE-Cy7, Live/Dead eFluor780. Cells were defined as follows: BMMO (CD11b^+^, F4/80^+^) and BMDC (CD11c^+^ MHCII intermediate to high).

Stained cells were washed twice with FACS buffer before overnight fixation with 2% PFA. Flow cytometry was run using LSRFortessa X-20 analyzer (BD Biosciences) using UltraComp beads (Invitrogen) for single color controls. All flow cytometry analyses were conducted using FlowJo (v.10.5).

### Syngeneic Mixed Lymphocyte Reaction (MLR)

*M. bovis* BCG-infected BMDC (3 × 10^4^ cells) were pulsed with 10 ug/mL of MHC-I restricted OVA peptide 257–264 or MHC-II restricted OVA peptide 323–339, and co-treated with either BG (MOI 1 or 5) or a normalized dose of LPS (22.5 or 112.5 EU/mL) for 24 h. Splenic T cells (Tc) from C57BL/6 OTI or OTII transgenic mice were isolated using Pan T cell Isolation Kit (Miltenyi Biotec, Germany) and labeled with 5 uM CellTrace Violet (ThermoFisher Scientific). Labeled T cells were incubated with BMDC at a BMDC:Tc ratio of 1:10 for 96 h. Cells were stained with the following antibody panel for T cells: Thy1.2 APC, CD4 PE, CD8 AlexaFluor488, CD62L BV605, CD44 BUV737, and Live/Dead eFluor780, before overnight fixation. T cells were defined as follows: CD4 T cells (Thy1.2^+^, CD4^+^) and CD8 T cells (Thy1.2^+^, CD8^+^). Flow cytometry was ran using LSRFortessa X-20 analyzer (BD Biosciences) using UltraComp beads (eBioscience) for single color controls. Division and proliferation indices were derived using FlowJo's proliferation modeling tool. Division index represents the average number of cell divisions undergone by the whole cell population and includes undivided cells; it is calculated as follows:

$$\begin{array}{l} Division\;index=\;\frac{Total\;number\;of\;divisions}{Initial\;number\;of\;cells}. \end{array}$$

Proliferation index is the total number of divisions divided by the number of dividing cells and excludes the undivided population:

$$\begin{array}{l} Proliferation\;index=\;\frac{Total\;number\;of\;divisions}{Number\;of\;dividing\;cells}. \end{array}$$

### Quantification of Cytokine Levels

BCG-infected or uninfected BMMO or BMDC (10^5^ cells) were incubated with BG at MOI 40 and 5, respectively or a normalized dose of LPS (300 and 37.5 EU/mL, respectively) for 24 h. ELISA was conducted on cell culture supernatants to determine the levels of TNFα, IFNγ, IL-6, IL-10, IL-12p40, and IL-12p70 using mouse ELISA kits (Invitrogen), according to the manufacturer's instructions.

### Nitric Oxide Determination

Nitrite oxide (NO) production in cell culture supernatants was quantified using Measure-iT high-sensitivity nitrite assay kit (ThermoFisher Scientific). Fluorescence readings were recorded at λ_ex/em_ 365/450 nm.

### RNA Sequencing and Functional Analyses

BMDC were treated with BG at MOI 5 or with a normalized dose of LPS (188 EU/mL) for 2 h, or left untreated before RNA extraction was conducted using QIAGEN RNeasy mini kit (Qiagen, Singapore). RNA integrity was checked using Agilent 4200 TapeStation System, and all samples had RNA integrity numbers ≥ 8.9. Libraries for RNASeq were made with TruSeq Stranded mRNA Library Prep Kit (Illumina) using 1–1.5 μg of RNA as input. Libraries were multiplexed and ran across two lanes on HiSeq 4000 Sequencing Systems (Illumina) to yield 2 × 151 bp paired-end reads with an average yield of ~55 million reads/sample. All raw sequences have been deposited in NCBI Sequence Read Archive (SRA) under BioProject number PRJNA544586.

FASTQ files (paired end; 2 × 151) were mapped using STAR aligner against the Genome Reference Consortium Mouse Build 38 (GRCm38). The mapped paired-end reads were counted using HTSeq (46) and unambiguously mapped read pairs were binned per gene. Gene-based read counts were further processed using Bioconductor package EdgeR (47). Briefly, gene counts were transformed into counts per million (CPM) reads and only genes with CPM value ≥1 across all samples were used in downstream analysis. Gene counts were then adjusted for library size and normalized using trimmed mean of m-values (TMM) method. Differential gene expression was calculated using the Fisher's exact test and genes were considered significantly differentially expressed if they met the cut off criteria, i.e., false discovery rate (FDR) ≤ 0.05 and log2 fold change ≥ 1. Differential gene expression data was visualized as heatmap using R Bioconductor package heatmap3 (https://cran.r-project.org/package=heatmap3).

Functional annotation was performed using DAVID (version 6.8) (48, 49) and the gene term enrichment for the significantly enriched annotation terms were extracted from the functional annotation chart. Annotation terms were considered significant if they had a fold enrichment ≥ 1.5 and -log_10_ FDR ≥ 1.

### Statistical Analyses

Statistical analyses were generated from Prism 7.0 (GraphPad, USA) and tests used are indicated in the figure legends. One-way and two-way ANOVA were conducted on experiments comparing across treatment groups under single and multiple conditions, respectively, with Holm-Sidak's correction as *post-hoc* test. Results with *p*-values <0.05 were defined as statistically significant.

## Results

### Immunomodulatory Properties of BG on Macrophages and Dendritic Cells

**Figure 1:**
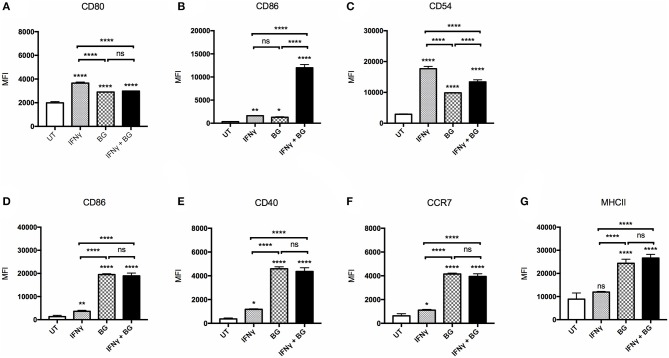
Activation status of BMMO and BMDC upon IFNγ and BG treatment. BMMO and BMDC were left untreated (UT), or were incubated with recombinant murine IFNγ at 20 and 10 μg/ml, respectively, or with BG at MOI 40 and 5, respectively, or with a combination of both for 24 h. Expression of surface activation markers CD80, CD86, CD54 on BMMO **(A–C)** and CD86, CD40, CCR7, and MHCII on BMDC **(D–G)** was determined by flow cytometry. Results are expressed as mean ± SD of technical triplicates and are representative of two independent experiments. Significance values were derived using 1-way ANOVA with Holm-Sidak's multiple comparisons test (ns: not significant, *p < 0.05, **p < 0.01, ****p < 0.0001); asterisks above bars indicates significance with respect to UT control.

The ability of BG to activate macrophages and dendritic cells was first examined. BMMO and BMDC were treated with BG at MOI 40 and 5, respectively, which corresponds to the maximum dose that did not lead to more than 20% cell death (data not shown). BG treatment resulted in a significant upregulation of all the activation markers tested (CD80, CD86, CD54, CD40, CCR7, MHCII) on bone marrow-derived macrophages (BMMO) (Figures 1A–C) and bone marrow-derived dendritic cells (BMDC) (Figures 1D–G) compared to untreated cells. However, the increased surface expression of CD80 and CD54 was significantly lower than that measured on BMMO treated with IFNγ alone (Figures 1A–C). In contrast, BG treatment led to significantly greater expression of all the activation markers on BMDC compared to treatment with IFNγ alone (Figures 1D–G). Notably, increased surface CCR7 expression on BG-treated BMDC suggested that BG might increase the migratory ability of DC (Figure 1F). When BMMO were co-incubated with both IFNγ and BG, significantly higher levels of CD86 and CD54 were measured compared to stimulation with BG alone, suggesting that IFNγ and BG may act synergistically to activate macrophages (Figures 1B,C). However, such synergy was not observed with BMDC (Figures 1D–G).

### BG Has Distinct Immunomodulatory Properties From LPS

**Figure 2:**
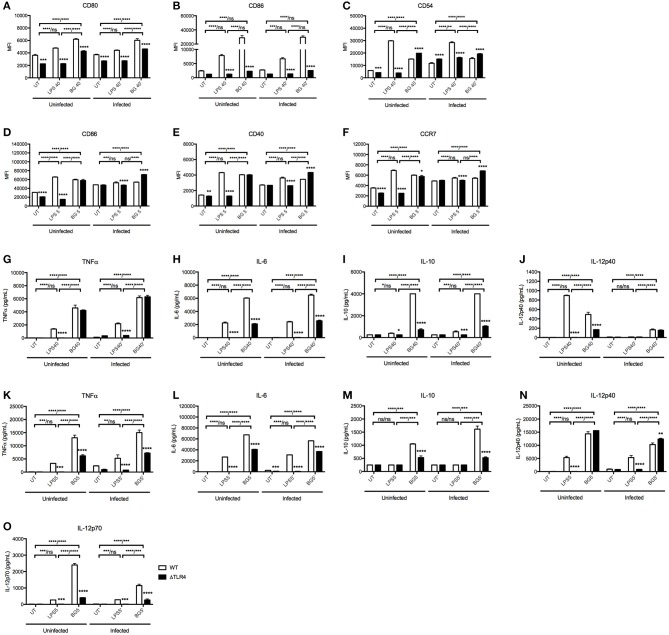
Activation status and cytokine production of WT vs. ΔTLR4 BMMO and BMDC upon BG and LPS treatment. Uninfected and *M. bovis* BCG-infected BMMO and BMDC were treated with BG at MOI 40 and 5, respectively, or with a normalized amount of LPS (1,500 and 188 EU/mL, respectively) or left untreated (UT) for 24 h before analysis of surface activation markers by flow cytometry. Expression levels of CD80, CD86, CD54 for BMMO **(A–C)** and CD86, CD40, and CCR7 for BMDC **(D–F)** are shown for WT (open bar) and ΔTLR4 (black bar) cells. Cell culture supernatants were collected to quantify the levels of TNFα, IL-6, IL-10, IL12p40, and IL-12p70 produced by ELISA. Cytokine production was measured in WT and ΔTLR4 BMMO **(G–J)** and BMDC **(K–O)**. Results are expressed as mean ± SD of technical triplicates **(A–F)** or duplicates **(G–O)** and are representative of two independent experiments. Significance values were derived using 2-way ANOVA with Holm-Sidak's multiple comparisons test (ns: not significant, *p < 0.05, **p < 0.01, ***p < 0.001, ****p < 0.0001). Asterisks separated by a dash denote significance values between treatment groups within WT and KO samples, respectively (WT/KO), while those above bars indicate significance with respect to WT control. Dotted lines denote limit of detection.

Since LPS is a major component of *E. coli* bacterial cell wall, we questioned whether LPS alone could recapitulate the immunostimulatory properties of BG. We thus compared the activation level of BMMO and BMDC treated with BG or with an amount of LPS equivalent to that present in the BG dose (see Methods), referred to as “normalized amount of LPS” thereafter. Furthermore, since LPS immunostimulatory properties signal mainly through TLR4 (50), we examined the ability of BG to activate TLR4-deficient BMMO and BMDC. The results indicated that BG activated BMMO and BMDC to a greater extent than a normalized amount of LPS, as evidenced by higher expression of most activation markers in both *M. bovis* BCG-infected and uninfected contexts (Figures 2A–F). BG- and LPS-mediated activation were found to be partially dependent on TLR4: ΔTLR4 BMMO displayed lower surface expression of CD80 and CD86 compared to WT BMMO (Figures 2A,B). In contrast, the reduced expression of CD54 in ΔTLR4 BMMO was seen only upon LPS treatment, implying that BG-induced upregulation of CD54 expression is TLR4-independent (Figure 2C). In BMDC, BG-mediated upregulation of CD86, CD40, and CCR7 was largely independent of TLR4, unlike LPS treatment (Figures 2D–F).

Cytokine production in culture supernatant was also measured in BG- and LPS-treated WT vs. ΔTLR4 BMMO and BMDC (Figures 2G–O). BG treatment generally induced higher levels of TNFα, IL-6, and IL-10 compared to LPS treatment in both uninfected and *M. bovis* BCG-infected BMMO (Figures 2G–J); similar results were obtained with BMDC, including IL-12p70, an important Th1 cytokine (Figures 2K–O). Interestingly, production of IL-12p40 in uninfected BMMO was greater upon LPS stimulation compared to BG treatment (Figure 2J), while the converse was observed in BMDC (Figure 2N); this suggests that production of this cytokine subunit is mediated by different signaling pathways in both cell types. Production of IL-12p70 instead was found TLR4-dependent upon stimulation with both LPS and BG (Figure 2O). Furthermore, LPS-induced production of all tested cytokines was undetectable in ΔTLR4 BMMO and BMDC, whereas BG treatment resulted in no or partial decrease of these cytokine levels in the absence of TLR4.

Overall, the data demonstrates that BG treatment of BMMO and BMDC results in activation patterns and cytokine production profiles that differ from those observed upon LPS treatment. Moreover, unlike LPS, BG-mediated cytokine production in BMMO and BMDC is partially TLR4-dependent, supporting BGs' ability to trigger alternative signaling pathways in these innate immune cells.

### BG-Induced NO Production Correlates With Enhanced Bacterial Killing in Macrophages

**Figure 3:**
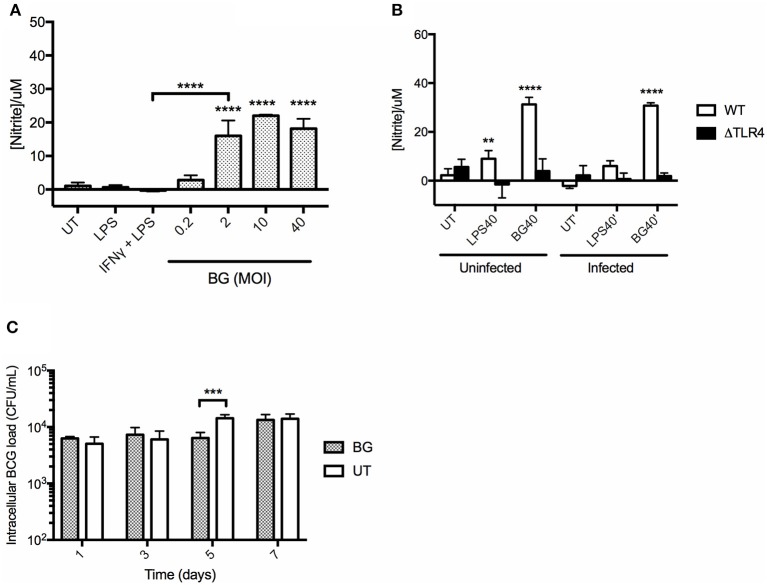
NO production in BMMO upon BG and LPS treatment and killing efficacy. **(A)** BMMO were left untreated (UT), or treated with 10 ng/mL LPS, or with a combination of (20 ng/mL IFNγ +10 ng/mL LPS), or with a range of BG MOIs for 24 h before nitrite determination. **(B)** Uninfected or *M. bovis* BCG-infected WT and ΔTLR4 BMMO were treated with BG at MOI 40 (BG40) or with a normalized amount of LPS (LPS40, corresponding to 1,500 EU/mL) or left untreated (UT) for 24 h before nitrite quantification. **(C)** *M. bovis* BCG-infected BMMO were treated with BG (MOI 40) or left untreated (UT). Intracellular bacterial loads were determined at 1, 3, 5, and 7 days post-infection. Results are expressed as mean ± SD of technical triplicates and are representative of two independent experiments. Significance values were derived using 1-way ANOVA for **(A)** and 2-way ANOVA for **(B,C)** with Holm-Sidak's multiple comparisons test (**p < 0.01, ***p < 0.001, ****p < 0.0001); asterisks above bars indicates significance with respect to UT **(A,C)** and WT **(B)** control.

BGs' ability to activate macrophages suggested their propensity to produce NO, an important mycobactericidal element. To test this hypothesis, BMMO were treated with a range of BG MOIs or with positive controls including (i) 10 ng/mL LPS and (ii) a combination of (20 ng/mL IFNγ + 10 ng/mL LPS). A significantly greater production of NO was measured in BMMO treated with BG at MOI 2 and above, compared to untreated BMMO, and BMMO treated with LPS or (IFNγ + LPS) (Figure 3A). In comparison to a normalized amount of LPS, BG exhibited a superior ability to trigger NO production in both *M. bovis* BCG-infected and uninfected contexts (Figure 3B). Furthermore, BG treatment of ΔTLR4 BMMO resulted in NO levels that were close to the detection limit (Figure 3B), indicating that BG-induced NO production is largely TLR4-dependent. Importantly, BG-mediated NO production in BMMO correlated with a transient but significant decrease in the intracellular bacterial load at day 5 post-infection (p.i.) (Figure 3C). This indicates that NO-dependent mechanisms may contribute to improved killing in BG-treated macrophages.

### Gene Expression Profile in BG-Treated and LPS-Treated Dendritic Cells

**Figure 4:**
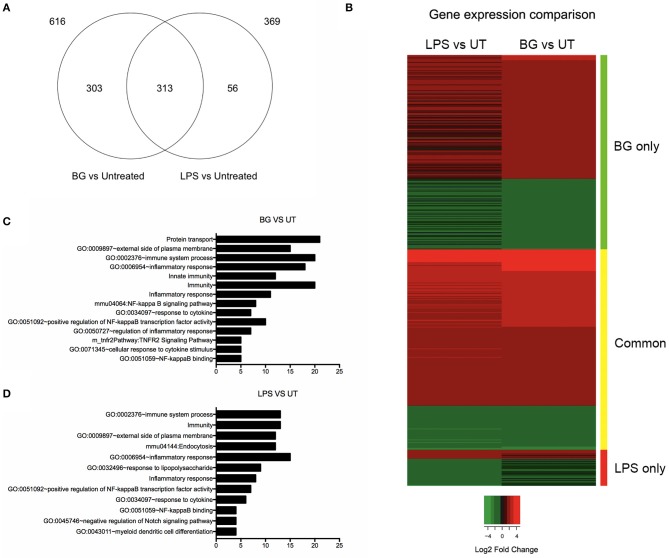
Comparison of differential gene expression profiles of BG- and LPS-treated BMDC. BMDC were treated with BG MOI 5 or a normalized amount of LPS (188 EU/mL) for 2 h before total RNA was extracted and processed for RNA sequencing. **(A)** Venn diagram shows the differentially expressed gene counts between BG and LPS treatment relative to untreated (UT) controls 2 h post-treatment. 369 and 616 genes were found differentially modulated from untreated (UT) controls in consensus between two independent experiments upon BG and LPS treatment, respectively. 313 genes were found commonly modulated between both treatments, while 56 and 303 genes were BG- and LPS-specific, respectively. **(B)** Heat map representation of differentially expressed genes for the conditions: BG and LPS relative to UT controls. Color panel above the heat map indicate the three sides of the comparison, i.e., green: significant in BG only, yellow: common in both, and red: significant in LPS only. Legend indicates log2 fold change, where green indicates down-regulation and red, up-regulation relative to UT. **(C,D)** Bar charts showing total number of genes within manually curated subsets containing immunologically relevant, significant gene terms (fold enrichment ≥ 1.5 and –log_10_ FDR ≥ 1.0) derived from functional annotation of differentially expressed genes upon BG **(C)** and LPS **(D)** treatment relative to UT.

To compare the overall impact of BG vs. LPS treatment on BMDC, a transcriptomics (RNAseq) approach was carried out. The number of genes that were differentially modulated by BG treatment compared to untreated BMDC was significantly greater than upon LPS treatment (616 vs. 369 genes, respectively) (Figure 4A). Most of the genes induced by LPS were found in common with BG treatment, leaving 56 genes unique to LPS treatment (Figure 4A, Tables S1, S2), whereas 303 genes were uniquely induced by BG treatment (Figure 4A, Table S3). Heat map analysis showed that BG treatment modulated gene expression differently from LPS (Figure 4B). Functional annotation analysis revealed that immune system processes involving both the innate and adaptive immune responses were commonly modulated upon both LPS and BG treatment (Figures 4C,D): these genes include *Bcl10, CD86, IL-4, Icam1* (CD54), *IL-27, CCL22*, and *CD14*, which facilitates TLR4-mediated responses (Tables S4, S5). Unlike treatment with a normalized amount of LPS, BG treatment significantly modulated *Nfkb1* gene expression, which supports the superior immune activation potential of BG. BG treatment was also found to influence a greater variety of pathways and corresponding gene counts than LPS (Figures 4C,D). Interestingly, only LPS treatment modulated the pathway GO:0032496 (response to lipopolysaccharide); BG may contain other components that dampen the LPS pathway response in favor of others (Figures 4C,D). Additionally, BG-induced modulation of *Lyst*, a lysosomal trafficking regulator, supports DC internalization and processing of BG (Tables S4, S5). Overall, the RNAseq data confirm the superior and broader modulatory ability of BG in comparison to a normalized amount of LPS.

### BG Modulates T Cell Proliferation and Differentiation *in vitro*

The ability of BG to increase the surface expression of co-stimulatory molecules CD80 and CD86 on BMDC, essential for T cell receptor binding, led us to investigate whether BG could promote T cell differentiation and proliferation. A syngeneic mixed lymphocyte reaction (MLR) assay was set up whereby uninfected or *M. bovis* BCG-infected BMDC were stimulated with OTI or OTII OVA peptide in the presence of BG or a normalized amount of LPS or in the absence of both, before being co-cultured with corresponding transgenic OTI and OTII T cells. T cell differentiation and proliferation, as well as cytokine production in the culture supernatants, were then measured.

Division and proliferation indices indicated that in the OTI system, comparable CD8 T cell proliferation was observed with BG-treated, LPS-treated and untreated BMDC, suggesting that BG treatment did not significantly impact on CD8 T cell proliferation (Figures S1A,B). However, BG treatment of BMDC resulted in a greater proportion of activated CD8 T cells compared to untreated and LPS-treated controls (Figure S1D). Interestingly, compared to untreated control, LPS or BG treatment of BMDC resulted in significantly lower proportion of naive (Figure S1C) as well as CD62L CD44 double positive CD8 T cells (Figure S1E), representative of central memory T cells (also CCR7^+^). As for cytokine production, OTI T cells co-cultured with BG-treated BMDC produced significantly greater levels of IFNγ and similar levels of IL-12p40 compared to untreated and LPS-treated conditions (Figures S1F,H). Expectedly, IL-4, which is mainly produced by CD4 T cells, was lowly/not detected in all the groups (Figure S1G).

**Figure 5:**
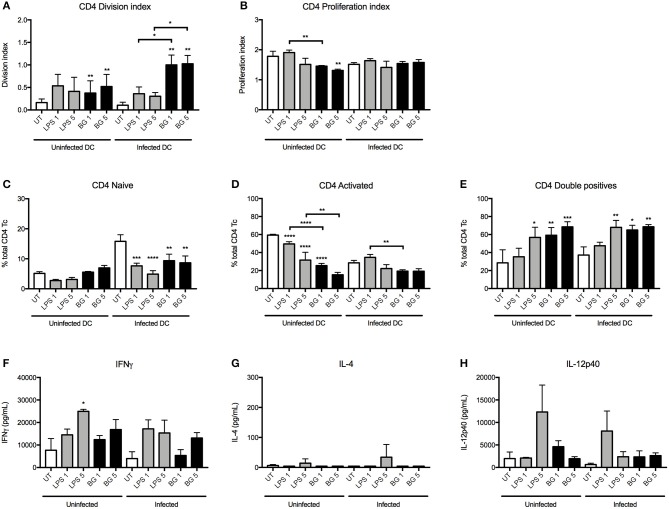
CD4 T cell proliferation and cytokine production induced by LPS- and BG-treated DC in a mixed lymphocyte reaction. *M. bovis* BCG-infected or uninfected BMDC were left untreated (UT) (open bars), stimulated with BG (MOI 1 and 5) (black bars) or stimulated with a normalized amount of LPS (23 or 113 EU/mL, respectively) (gray bars) in the presence of OTII OVA peptide for 24 h, before co-culture with transgenic T cells for 96 h. Division and proliferation indices of CD4 **(A,B)** T cells were derived. Proportion of naïve (CD62L^+^ CD44^−^), activated (CD62L^−^ CD44^+^) and double positive T cells were displayed as percentages of total CD4 T cells **(C–E)**. The levels of IFNγ, IL-4, and IL-12p40 produced after 96 h incubation were measured **(F–H)**. Results are expressed as mean ± SD of technical triplicates and are representative of two independent experiments. Significance values were derived using 1-way ANOVA with Holm-Sidak's multiple comparisons test (*p < 0.05, **p < 0.01, ***p < 0.001, ****p < 0.0001); asterisks above bars indicate significance with respect to UT control. Dotted lines denote limit of quantification.

In the OTII system, the division index indicated that BG-treated BMDC induced a greater proportion of proliferating CD4 T cells in both the uninfected and infected contexts compared to untreated controls (Figure 5A). Moreover, BG treatment of infected BMDC resulted in a significantly higher proportion of CD4 T cells to undergo cell division compared to LPS treatment. The extent of proliferation was generally comparable or lower between BG-treated BMDC and the other groups (Figure 5B). Interestingly, BG treatment resulted in reduced proportion of activated CD4 T cells and a greater proportion of CD62L CD44 double positive CD4 T cells compared to untreated controls (Figures 5C–E). In term of cytokine production, similar levels of IL-12p40 were measured in BG-treated and LPS-treated groups (Figure 5H). Furthermore, slightly elevated levels of IFNγ and very low levels of IL-4 were obtained with BG-treated BMDC compared to untreated controls (Figures 5F,G), suggesting that BG treatment of BMDC promotes T cell differentiation into CD4 Th1 lineage.

Overall, the data obtained from these MLR experiments demonstrated that BG treatment of BMDC significantly influences the proliferation and/or differentiation of naïve T cells. BG-treated BMDC generally led to greater IFNγ production by both CD4 and CD8 T cells, which suggests that BG treatment may help to control mycobacterial replication more effectively.

### BG Treatment Influences the Composition of Lung Immune Cell Populations and Cytokine Production in *M. bovis* BCG-Infected Mice

**Figure 6:**
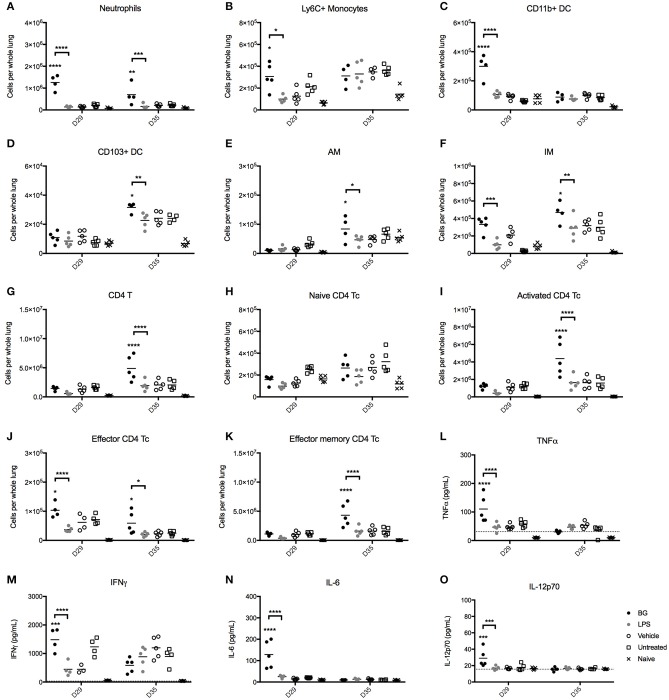
Immune cell populations and cytokine production in lungs from BG-treated vs. LPS-treated *M. bovis* BCG-infected mice. Adult female C57BL/6 mice were infected intratracheally (IT) with 10^6^ CFU of *M. bovis* BCG. Starting from day 7 post-infection, mice were administered IT weekly and for 4 consecutive weeks with 10^6^ BG, a normalized amount of LPS (75 EU), vehicle or were left untreated (UT). Lungs were harvested 1 day (day 29 p.i.) or 1 week (day 35 p.i.) after the last dose administered and the myeloid **(A–F)** and CD4 T cell **(G–K)** populations were analyzed by flow cytometry. Levels of TNFα, IFNγ, IL-6, IL-10, IL12p40, and IL-12p70 in the lung homogenates **(L–O)** were determined by ELISA. Results are representative of two independent experiments and data points are shown for each mouse (*n* = 4–5 mice/group). Significance values were derived using 2-way ANOVA with Holm-Sidak's multiple comparisons test (*p < 0.05, **p < 0.01, ***p < 0.001, ****p < 0.0001); asterisks above bars indicate significance with respect to vehicle control. Dotted lines denote limit of quantification. pDC, plasmacytoid DC; AM, alveolar macrophages; IM, interstitial macrophages.

The immunostimulatory properties of BG were next examined *in vivo*. The impact of BG and a normalized dose of LPS on pulmonary cell populations was examined in an established *M. bovis* BCG infection mouse model in C57BL/6 mice. Starting at day 7 p.i., BG and LPS were administered intratracheally to *M. bovis* BCG-infected mice once weekly for 4 consecutive weeks. Pulmonary innate immune cell populations were analyzed at 1 day (day 29 p.i.) or 1 week (day 35 p.i.) after the last BG or LPS treatment dose. The number of neutrophils and interstitial macrophages (IM) was significantly higher at both time points in the BG-treated group compared to the other groups, which may suggest that both innate immune cell populations may play a significant role in mycobacteria killing. In contrast, increased levels of Ly6C^+^ monocytes were seen only at day 29 p.i. (Figures 6A,B,F). Elevated levels of CD11b^+^ DC, which are typically responsible for T cell activation (51) were also measured 1 day post-BG treatment (Figure 6C). Lung resident CD103^+^ DC, which acquire apoptotic cells and traffic to the draining lymph nodes (51), also displayed increased levels in BG-treated animals at day 35 p.i. (Figure 6D), which may reflect a greater antigen presentation activity in the lungs of these mice. Levels of resident alveolar macrophages (AM) were also raised at day 35 p.i. upon BG treatment (Figure 6E); this is in line with their role in protection of lung tissue from destruction by inflammatory mediators or damaging oxidative burst and in recruitment of monocyte-derived macrophages (52, 53). Interestingly, treatment with a normalized dose of LPS did not lead to any significant changes in the number of all these innate immune cell populations compared to untreated or vehicle-treated infected mice (Figures 6A–F).

We also analyzed the CD4 and CD8 T cell populations present in the lungs from BG- and LPS-treated *M. bovis* BCG-infected mice 1 day (day 29 p.i.) or 1 week (day 35 p.i.) after the last treatment dose. The total CD4 T cell count was significantly increased in BG-treated mice 1 week after the last treatment dose, compared to the other treatment groups (Figure 6G). This correlated with an increased number of activated CD4 T cells (Figure 6I), and effector and effector memory CD4 T cells (Figures 6J,K) compared to the other groups. These observations thus showed that BG treatment augmented the number of both short-lived CD4 effectors and transitional CD4 effector memory cells that possess rapid effector functions, including production of IFNγ and IL-4 (54). On the other hand, and consistent with our *in vitro* observations, BG treatment impacted minimally on the CD8 T cell populations, as evidenced by comparable levels of CD8 T cell subsets across all the infected groups (Figures S2A–E). A mild but significant increase in the number of CD8 effectors was nevertheless observed 1 day after the last BG dose, although this was not sustained after 1 week (Figure S2D). Again, LPS treatment did not significantly affect the CD4 and CD8 T cell populations, underscoring the superior immunostimulatory properties of BG over LPS.

Finally, the levels of key cytokines in the lung homogenates of BG-treated vs. LPS-treated mice were quantified. One day after the last treatment dose (day 29 p.i.), BG-treated animals displayed significantly higher levels of TNFα, IFNγ, IL-6, and IL-12p70 compared to those measured in vehicle-treated and untreated infected mice (Figures 6L–O), supporting the pro-inflammatory effect of BG. In contrast, LPS-treated infected mice did not display enhanced levels of pro-inflammatory cytokines compared to controls. No significant difference was found among the treatment groups for all cytokines tested 1 week after the last treatment dose (day 35 p.i.).

### BG Treatment Reduces Lung Bacterial Loads in *M. bovis* BCG- and Mtb-Infected Mice

**Figure 7:**
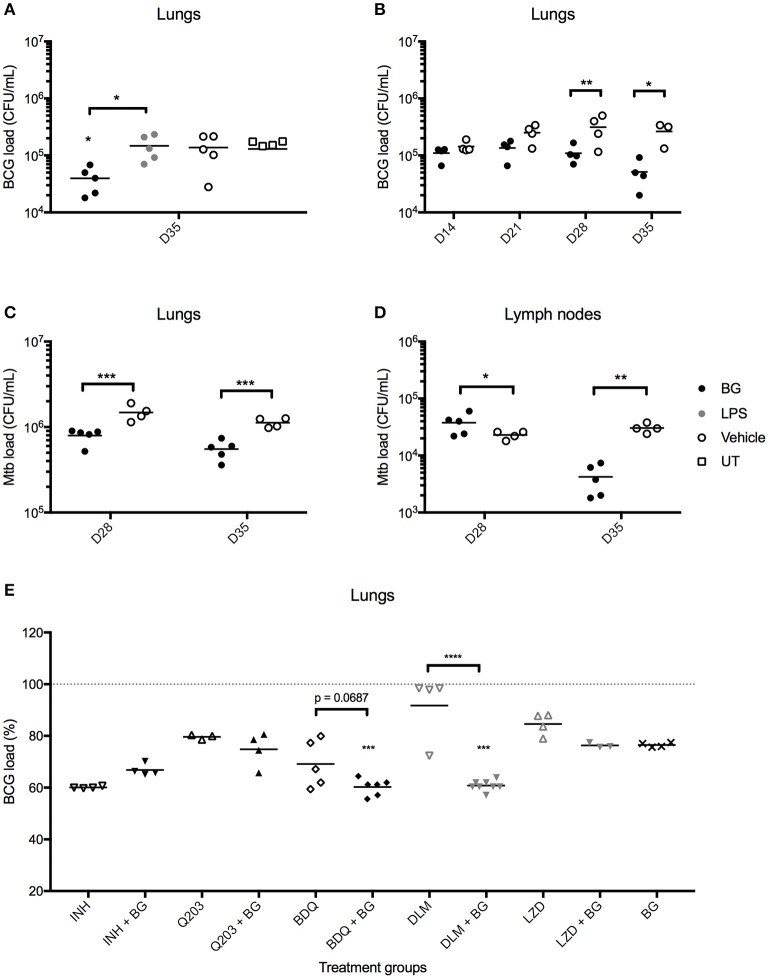
Effect of BG treatment on lung bacterial loads in *M. bovis* BCG- and Mtb-infected mice. Adult female C57BL/6 mice were infected intratracheally (IT) with 10^6^ CFU of *M. bovis* BCG **(A,B,E)** or 10^3^ CFU Mtb H37Rv **(C,D)**. Treatment was administered as described in the legend of Figure 6. For BG adjunct therapy, infected mice were treated daily with INH (0.05 mg/mL) or thrice weekly with Q203 (10 mg/kg) BDQ (15 mg/kg), DLM (10 mg/kg) or LZD (50 mg/kg) via the oral route, in combination with IT administration of BG as described in the legend of Figure 6. Organs were harvested at day 35 p.i., unless otherwise indicated, and homogenates were plated for CFU enumeration. Data points are shown for each mouse (*n* = 3–8 mice/group) and *M. bovis* BCG load **(E)** is expressed as a percentage of vehicle control. Significance values were derived using 1-way **(E)** or 2-way **(A–D)** ANOVA with Holm-Sidak's multiple comparisons test (*p < 0.05, **p < 0.01, ***p < 0.001, ****p < 0.0001); asterisks above data points indicate significance with respect to vehicle control. Dotted lines denote percentage of *M. bovis* BCG load of vehicle control.

We next questioned whether the greater recruitment of immune cells observed in the lungs of BG-treated infected mice correlated with enhanced bacteria killing efficacy. Strikingly, BG-treated mice but not LPS-treated mice displayed significantly lower pulmonary bacterial loads at day 36 post-infection (1 week after the last treatment dose) compared to vehicle and untreated groups (Figure 7A). Both treatments were well-tolerated across all groups, with both BG- and LPS-treated groups displaying a body weight change profile similar to vehicle-treated and untreated groups (Figure S3A). Histological analysis of lung sections examined at day 36 p.i. indicated no significant difference in BG- and LPS-treated animals compared to vehicle and untreated controls, implying that both treatments did not cause overt inflammation, nor did they adversely affect lung architecture (Figure S3B). To further characterize the BG-mediated effects, the bacterial loads were determined at day 14, 21, 28, and 35 p.i. A significant reduction in bacterial loads was observed at day 28 and 35 p.i. in the lungs from BG-treated mice compared to vehicle-treated controls (Figure 7B). Importantly, similar observations were made in Mtb-infected mice whereby a significant reduction in pulmonary bacterial loads was observed at day 28 and 35 p.i. in BG-treated mice (Figure 7C). It is worthy to note that reduction in bacterial burden started to be seen at day 28 p.i., which coincides with the activation of adaptive immunity. This suggests that the effector mechanisms responsible for improved mycobacterial killing involve the adaptive immunity. Interestingly, BG treatment significantly diminished the bacterial load in lymph nodes at day 35 p.i., 1 week after the last BG dose was administered in Mtb-infected mice (Figure 7D), but not in *M. bovis* BCG-infected ones (data not shown). This observation may suggest that BG immune stimulatory effects are greater in the context of infection with more virulent pathogens that may sensitize the host immunity to additional stimuli. Although the decrease in lung bacterial loads was sustained up to 1 week after the last BG dose (Figures 7B,C), it did not extend beyond 3, 4, and 5 weeks after the last BG dose—day 42, 49, and 56 p.i., respectively (Figure S3C). This latter observation indicates that weekly BG administration is necessary to sustain reduced pulmonary bacterial loads. Additionally, single dose BG treatment administered at day 7 p.i., or at day 21 or 28 p.i. (when adaptive immunity has been activated) did not lead to significantly lower pulmonary bacterial loads at day 35 p.i. (Figure S3D). Neither did 2 weekly BG doses administered at day 21 and 28 p.i. (Figure S3D). These observations thus suggest that a regimen of at least 4 BG weekly doses is necessary to achieve significant reduction in bacterial load.

### BG Treatment Synergizes With Second-Line TB Drugs

Given its potential in reducing mycobacterial load, BG treatment was evaluated in combination with selected anti-TB compounds. Since second-line drugs possess comparatively lower cidal potential and higher toxicity than their first-line counterparts, the potential synergistic killing effect between BG and various second-line agents bedaquiline (BDQ), delamanid (DLM) and linezolid (LZD) was examined *in vivo*. First-line drug isoniazid (INH), and phase II clinical drug candidate Q203 were also included. Oral dosing regimens were adapted from established drug regimens for each drug (55–57) to achieve a sub-optimal killing in order to observe any synergistic cidal effect when co-treated with BG. The results indicated that lower pulmonary bacterial loads were observed with BDQ + BG and DLM + BG groups compared to drug only-treated groups (Figure 7E). In contrast, BG treatment did not enhance killing efficacy in INH-, Q203-, and LZD-treated mice. Together, the data showed that BG synergizes with at least two second-line TB drugs DLM and BDQ.

## Discussion

Our work describes the immunostimulatory properties of BG in the context of mycobacterial infection, and demonstrates that pulmonary delivery of BG helps reduce lung mycobacterial loads. The significant reduction in bacterial loads in the lungs from BG-treated *M. bovis* BCG- and Mtb-infected mice correlated with increased numbers of key innate (neutrophils, macrophages, dendritic cells) and adaptive (CD4 effector cells) immune cell populations and elevated levels of key Th1 cytokines (TNFα, IFNγ, IL-12p70). Hence, BG treatment has the potential to improve the killing activity of innate immune cells and tip the Th1/Th2 balance toward the more protective Th1 response.

These *in vivo* observations were consistent with our *in vitro* data showing that BG effectively activated both macrophages and DC, as evidenced by the increased expression of surface activation markers—CD80/86, CD54, and MHCII—and increased production of TNFα, IL-6, IL-10, and IL-12p40. This finding is in agreement with previous BG stimulation studies conducted on murine BMDC (58), bovine monocyte-derived DC (59), and THP-1 macrophages (60). We also propose that BG may increase the migratory potential of DC through upregulation of CCR7, essential for activation of naïve T cells in the draining lymph nodes. Furthermore, in line with previous studies (45, 59, 61) our MLR experiments showed that BG-treated DC resulted in enhanced CD4 T cell proliferation and differentiation. Unlike previous studies however (38, 41, 45), we failed to observe a significant impact of BG treatment on CD8 T cell proliferation both *in vitro* and *in vivo*. Nevertheless, an increased proportion of activated CD8 T cells supports that BG treatment does potentiate CD8 T cells in their effector functions.

Consistent with a previous report (43), BG-activated macrophages were proficient in producing NO, the importance of which has been well-documented in controlling TB infection. iNOS knockout mice were found to be more susceptible to Mtb infection (62). Moreover, in TB patients, RNIs are capable of inactivating essential pathogenic components that limit mycobacterial persistence within the host (63, 64). The strong induction of NO production upon BG treatment may thus help counteract the limited amount of RNI produced during Mtb infection. However, only a moderate and transient reduction in intracellular bacterial loads was seen in *M. bovis* BCG-infected BMMO treated with BG. Thus, the main mechanism by which BG treatment leads to reduced mycobacterial loads in murine lungs may not predominantly rely on increased NO production. This hypothesis is further supported by the delayed effect of BG treatment on the pulmonary mycobacterial loads *in vivo*, that was observed only at day 28 and 35 p.i., but not at earlier time points, despite BG treatment commencing at day 7 p.i. Our data thus suggest that BG treatment impacts the adaptive immunity (in particular CD4 T cells) through potentiating innate immune cells (DC and macrophages mainly). The fact that several weekly BG doses were required to observe a significant reduction in the bacterial loads points at trained innate immunity, although this is purely speculative at this stage. Furthermore, the effect was not sustained beyond 1 week after the last BG dose, implying that continued BG dosing is necessary to maintain stimulation of both the innate and adaptive immunity.

Importantly, our work clearly demonstrates that treatment with an equivalent amount of purified LPS that is present in the BG dose could not recapitulate the effects seen with BG treatment. This was especially apparent *in vivo*, where LPS treatment of *M. bovis* BCG-infected mice did not influence the lung immune cell populations, cytokine levels and bacterial burden compared to infected controls. Consistently, we demonstrated that BG-induced innate immune activation is only partially TLR4-dependent (with the exception of NO production, which was fully mediated by TLR4), and involved a broader and stronger intracellular signaling response compared to LPS, although the latter observation was not verified at the protein level. The presence of a variety of TLR ligands on BG likely explains the greater immunostimulatory potential of BG over LPS, without overt inflammation.

Additionally, BG treatment enhanced the *in vivo* bactericidal efficacy of second-line drugs—DLM and BDQ. Such synergistic killing was not observed for INH + BG. A previous study reported that host cell activation by nitrosative stress induces tolerance to first-line drugs, including INH, in Mtb (65). Since BG treatment induces NO production in murine macrophages, it is thus possible that BG-mediated activation of macrophages could compromise the killing efficacy of INH. The lack of improved killing in mice treated with Q203 + BG or LZD + BG may be attributed to the immunomodulatory abilities of these drugs that could hinder and/or counteract those of BG. While this latter hypothesis remains to be experimentally explored, our data indicate that BG treatment is not universally compatible with all anti-TB agents/compounds, but can improve the efficacy of certain second-line agents.

This work paves the way toward the possible use of BG as an adjunct TB immunotherapy, although much work remains to be done. Specifically, it will be very important to confirm our observations in TB models. In addition, the cellular and molecular mechanisms involved in BG-mediated therapeutic effects should be deciphered. Furthermore, as BG production is limited to Gram-negative bacteria (to allow formation of the transmembrane tunnel), it is not possible to produce BG from mycobacteria. However, *E. coli* BG may be further engineered with mycobacterial antigens expressed on their surface in order to trigger a TB-specific immune response and further enhance the therapeutic potential of BG treatment.

## Data Availability Statement

The datasets generated for this study can be found in the all raw sequences have been deposited in NCBI Sequence Read Archive (SRA) under BioProject number PRJNA544586.

## Ethics Statement

The animal study was reviewed and approved by the Institutional Animal Care and Use Committee of National University of Singapore (NUS) under protocol R15-1030. All the animal experiments were performed in the AALAAC-accredited animal facilities.

## Author Contributions

JL, GP, and SA designed the experiments. JL, VK, SC, MV, BP, and DC performed the experiments. JL, VK, BP, PD, and SA analyzed the data. PK and WL provided the reagents. JL and SA wrote the manuscript.

### Conflict of Interest

PK and WL were employed by Biotech Innovation Research Development & Consulting (BIRD-C). The remaining authors declare that the research was conducted in the absence of any commercial or financial relationships that could be construed as a potential conflict of interest.
