# Supplementary material for: Harnessing the Immunomodulatory Properties of Bacterial Ghosts to Boost the Anti-mycobacterial Protective Immunity
Source: Front Immunol. 2019 Nov 22;10:2737. doi: 10.3389/fimmu.2019.02737 (PMC6883722; doi:10.3389/fimmu.2019.02737)
Supplement: Supplementary file 1 [file Data_Sheet_1.docx]

**HARNESSING the immunomodulatory properties of bacterial ghosts TO boost the anti-mycobacterial protective immunity**

**Supplemental Material**

**Authors:** Jieling LIM^1,2^, Vanessa Hui Qi KOH^1,2^, Sharol Su Lei CHO^1,2^, Balamurugan PERIASWAMY^3^, Dawn Poh Sum CHOI^3^, Maurizio VACCA^1,2^, Paola Florez DE SESSIONS^3^, Pavol KUDELA^4^, Werner LUBITZ^4^, Giorgia PASTORIN^5^, and Sylvie ALONSO^1,2*^

^1^Department of Microbiology and Immunology, Yong Loo Lin School of Medicine, National University of Singapore, Singapore.

^2^Immunology programme, Life Sciences Institute, National University of Singapore, Singapore.

^3^Genome Institute of Singapore, Agency for Science, Technology and Research (A*STAR), Singapore.

^4^Biotech Innovation Research Development & Consulting (BIRD-C), Vienna, Austria. ^5^Department of Pharmacy, Faculty of Science, National University of Singapore, Singapore.

*Corresponding author: Immunology Programme, Centre for Life Sciences, 28 Medical Drive, NUS, Singapore 117456. Tel: +65 65163541; Email: [micas@nus.edu.sg](mailto:micas@nus.edu.sg); Fax: +65 67782684.

**Supplemental Tables (Excel File)**

**Table S1.** **List of differentially modulated genes found in common between LPS and BG treatment relative to UT controls.**

FDR ≤ 0.05 and log2 fold change ≥ 1. Genes listed are conserved between 2 independent experiments.

**Table S2.** **List of differentially modulated genes unique to LPS treatment relative to UT controls.**

FDR ≤ 0.05 and log2 fold change ≥ 1. Genes listed are conserved between 2 independent experiments.

**Table S3.** **List of differentially modulated genes unique to BG treatment relative to UT controls.**

FDR ≤ 0.05 and log2 fold change ≥ 1. Genes listed are conserved between 2 independent experiments.

**Table S4.** **List of significant genes derived from functional annotation of all differentially modulated genes upon LPS treatment**.

Fold enrichment ≥ 1.5 and -log_10_ FDR ≥ 1.0.

**Table S5.** **List of significant genes derived from functional annotation of all differentially modulated genes upon BG treatment.**

Fold enrichment ≥ 1.5 and -log_10_ FDR ≥ 1.0).

**Fig S1. CD8** **T cell proliferation and cytokine production induced by LPS- and BG-treated DC in mixed lymphocyte reaction**.

*M. bovis* BCG-infected or uninfected BMDC were left untreated (UT) (open bars), stimulated with BG (MOI 1 and 5) (black bars) or stimulated with a normalized amount of LPS (23 or 113 EU/mL respectively) (grey bars) in the presence of OTI OVA peptide for 24h, before co-culture with transgenic T cells for 96h. Division and proliferation indices of CD8 (A – B) T cells were derived. Proportion of naïve (CD62L+ CD44-), activated (CD62L- CD44+) and double positive T cells were displayed as percentages of total CD8 T cells (C – E). The levels of IFNγ, IL-4 and IL-12p40 produced after 96h incubation were measured (F – H). Results are expressed as mean ± SD of technical triplicates and are representative of two independent experiments. Significance values were derived using 1-way ANOVA with Holm-Sidak’s multiple comparisons test (*p<0.05, **p<0.01, ***p<0.001, ****p<0.0001); asterisks above bars indicate significance with respect to UT control. Dotted lines denote limit of quantification.

**Fig S2. CD8 T cell populations in lungs of BG-treated vs LPS-treated *M. bovis* BCG-infected mice.**

Adult female C57BL/6 mice were infected intratracheally (IT) with 10^6^ CFU of *M. bovis* BCG and treated with BG by IT as described in legend of Fig 6. Lungs were harvested one day (day 29 p.i.) or one week (day 35 p.i.) after the last dose administered and CD8 T cell populations (A – E) were analyzed by flow cytometry. Results are representative of two independent experiments and data points are shown for each mouse (n=4–5 mice/group). Significance values were derived using 2-way ANOVA with Holm-Sidak’s multiple comparisons test (*p<0.05, **p<0.01, ***p<0.001, ****p<0.0001); asterisks above bars indicate significance with respect to vehicle control.

**Fig S3. Effect of BG treatment on lung bacterial loads, body weight profile and lung histology of *M. bovis* BCG-infected mice.**

*M. bovis* BCG-infected adult female C57BL/6 mice were treated with BG, a normalized amount of LPS, vehicle or left untreated as indicated in the legend of Fig. 6. (A) Body weight was monitored daily upon commencement of BG/LPS treatment. Data are expressed as means ± SD (n=4 mice/group). Orange vertical lines indicate point at which treatment was administered. (B) At day 36 p.i., lungs were harvested from euthanized mice and fixed with formaldehyde before H&E staining. Lung sections are shown at 5X and 40X objectives (n=2 mice/group). (C) Lung bacterial loads of BG-treated and UT *M. bovis*-infected mice were determined at the indicated time points. (D) *M. bovis* BCG-infected mice were treated with BG at the indicated time point(s) before evaluating their pulmonary bacterial loads at day 35 p.i., expressed as a percentage of that in untreated control mice. Results are representative of two independent experiments and data points are shown for each mouse (n=4 mice/group). Significance values were derived using 1-way (D) or 2-way (C) ANOVA with Holm-Sidak’s multiple comparisons test. (*p<0.05, ****p<0.0001); asterisks above bars indicate significance with respect to UT.


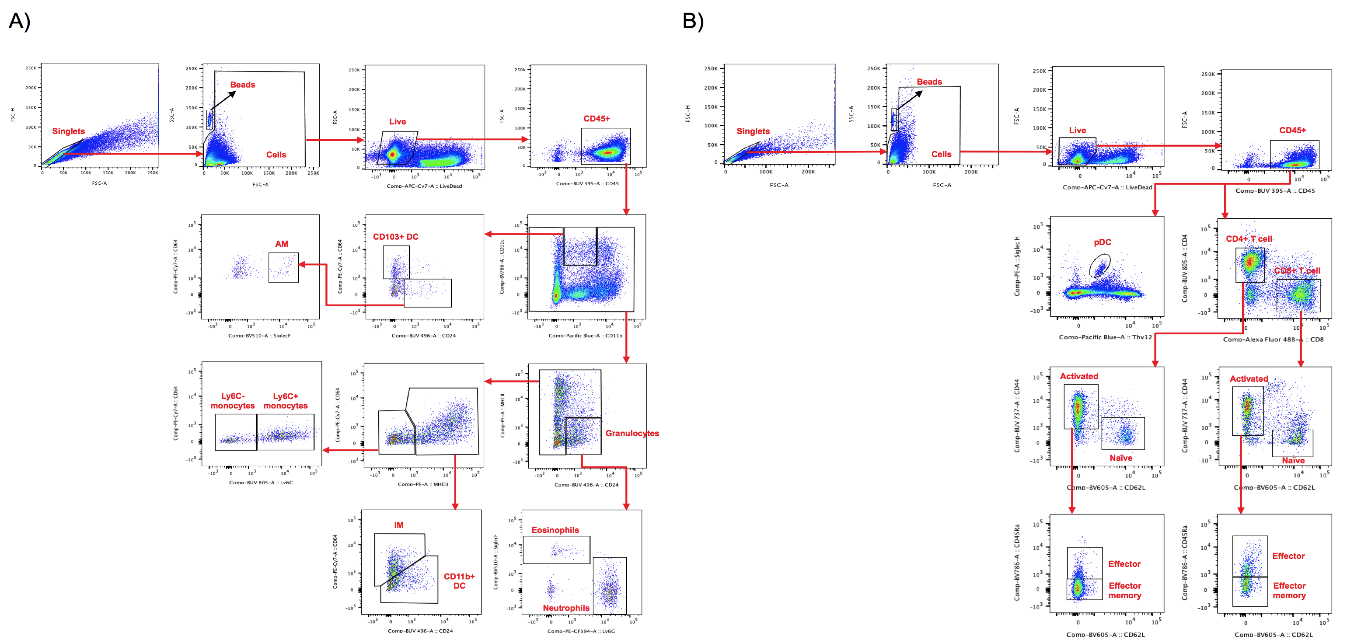


**Fig S4. Gating strategy used to define myeloid (A) and T cell (B) populations in the lungs of *M. bovis* BCG-infected C57BL/6 mice.**
